# Cavernous Angioma: A Rare Cause of Multiple Cranial Nerve Palsies

**DOI:** 10.7759/cureus.67464

**Published:** 2024-08-22

**Authors:** Kavyaashree Karthikeyan Meenakshi, Madhumitha S, C. H. Naga Sekhar, J Kumar, Krishnaswamy Madhavan

**Affiliations:** 1 Department of General Medicine, SRM Medical College Hospital and Research Centre, SRM Institute of Science and Technology (SRMIST), Kattankulathur, IND

**Keywords:** arteriovenous malformations, pontine hemorrhage, intracerebral hemorrhage, cavernous angioma, cranial nerve palsy

## Abstract

Acute onset of neurological deficit is highly suggestive of stroke; in such cases, computed tomography (CT) brain is the initial choice of investigation. While CT brain can differentiate between hemorrhagic and ischemic infarct, more often than not, it is unable to detect the underlying etiology of intracerebral hemorrhage. In these situations, magnetic resonance imaging (MRI) brain is crucial in determining the exact etiology and helps us tailor the specific management best suited for our patient. The case under discussion is of a 48-year-old male who presented with multiple cranial nerve palsies and ipsilateral cerebellar involvement in whom CT brain revealed a hemorrhage involving left hemipons and left middle cerebellar peduncle while an MRI brain revealed an unexpected cavernous angioma which changed the management and prognosis of the patient, proving its superiority over CT brain.

## Introduction

A cerebral cavernous angioma is a collection of aberrant blood vessels that are low-flow vascular malformations of the central nervous system; these are occult angiographically [1]. It is also referred as cerebral cavernous malformations, cavernoma, or cavernous hemangiomas [2]. A typical cavernous angioma looks like a raspberry or popcorn in T2-weighted sequences [1]. These malformations can manifest de novo following radiosurgery, familial or sporadically [3]. A single cavernous angioma typically manifests in sporadic cases, whereas 40%-60% of familial cases present as multiple cavernous angiomas that are autosomal dominant in inheritance [4]. Within the general population, cavernous angioma incidence ranges from 0.4% to 0.8% [5]. Cavernous angioma develops most frequently in the second to fifth decades of life [5]. Cavernous angioma most commonly occurs in the supratentorial region, where it commonly manifests as seizures or a focal neurologic deficit, while infratentorial cavernoma typically manifests as ataxia [6]. Early onset and female gender are linked to an increased risk of infratentorial cavernomas [5]. In our case, the patient had a bizarre presentation as he presented with multiple cranial palsies involving the pons and medulla oblongata along with involvement of the cerebellum, making it hard to localize to a particular region. There has been only a few articles with similar presentations published in the literature so far, making it a rarity [7-10].

## Case presentation

A 48-year-old male came to the outpatient department at 9 AM with complaints of giddiness since early morning; the patient was able to continue his routine activities despite the giddiness. Giddiness was acute in onset with no positional variation, aural fullness, vertigo, tinnitus, or gait disturbance. He had no known comorbidities and no similar family history. His systemic examination including central nervous system (CNS) examination, vitals, and electrocardiogram (ECG) was normal. Therefore, he was treated symptomatically with betahistine.

At 4 PM, he came to the casualty with complaints of numbness over the left side of his face, and his neurological examination revealed decreased sensation in the ophthalmic, mandibular, and maxillary nerve divisions of the trigeminal nerve on the left side of the face, but the motor component of the trigeminal nerve was uninvolved. His consensual reflex was normal. He had no cerebellar signs and no motor deficit at that point in time.

Two hours later at 6 PM, he complained of inability to close his left eye, and on examination, he had lagophthalmos in the left eye, loss of wrinkle on the left side of the forehead, loss of nasolabial fold in the left, and deviation of the angle of the mouth to the right.

Three hours later at 7 PM, the CNS examination of the patient revealed nystagmus in primary gaze and bidirectional nystagmus. Since the patient had nystagmus even in the primary gaze, ocular movements appeared to be restricted while there was no apparent paralysis of ocular movements. On the finger-nose test, he had dysmetria bilaterally. He was swaying to the left while walking.

Later at 8 PM, he developed slurring of speech and difficulty in swallowing both liquid and solids and nasal regurgitation of food, and his CNS examination revealed dysarthria and an absent gag reflex. He had no sensory deficit over his limbs and trunk. Bilaterally, plantar reflex, deep tendon reflexes, and fundi examination were normal. The patient was conscious and oriented to time, place, and person throughout the hospital stay. His vitals and cardiac and respiratory auscultation were also normal throughout the hospital stay.

**Figure 1:**
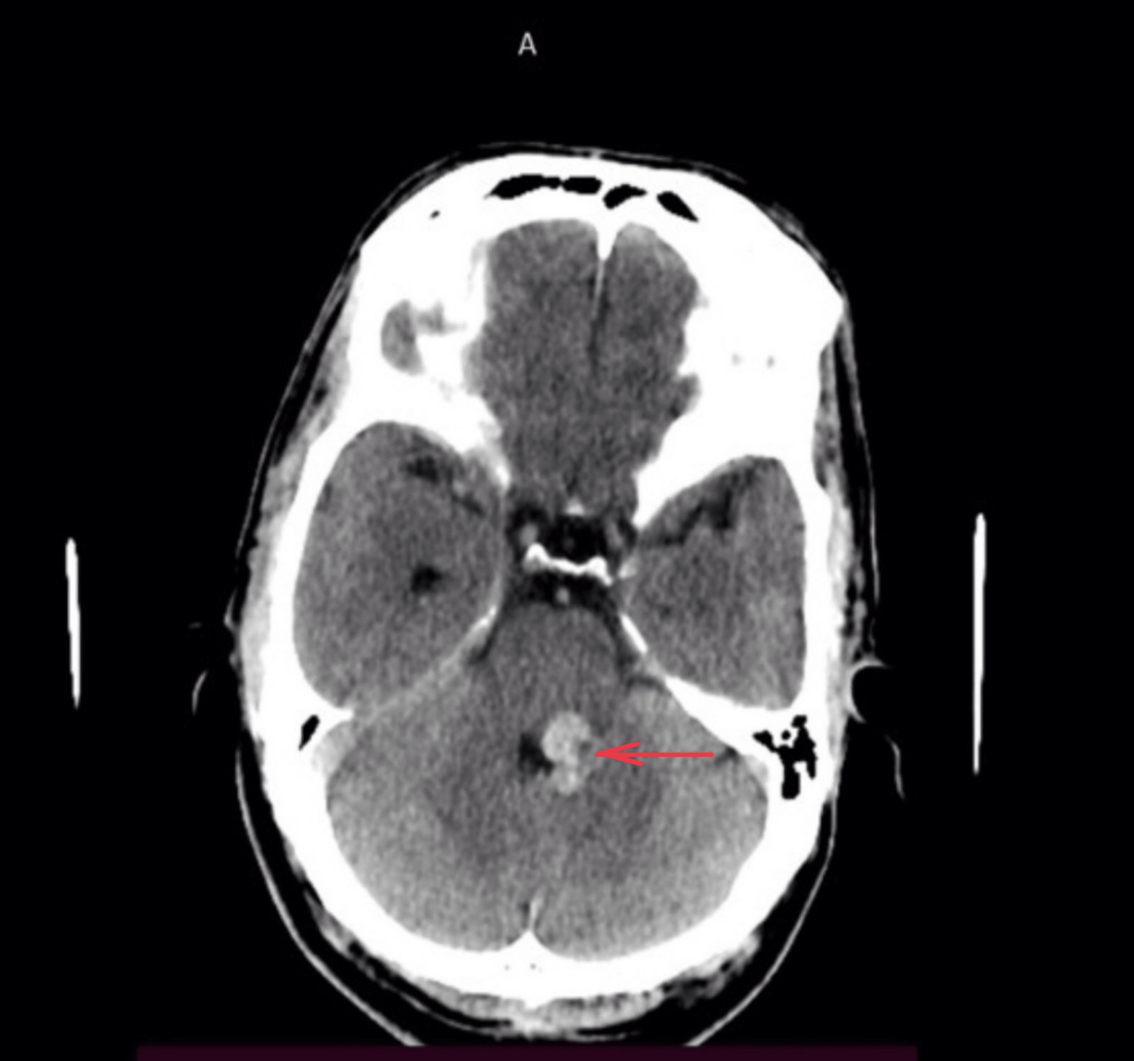
CT brain showing hyperdense lesion involving the left hemipons and left middle cerebellar peduncle indicating a hemorrhage pointed using red arrow CT: Computed tomography

Based on his symptoms and examination findings, it was found that the sensory nuclei of the left trigeminal nerve, left lower motor neuron facial nerve palsy, glossopharyngeal nerve, vagus nerve, and ipsilateral cerebellum were involved. The motor components of the trigeminal nerve, oculomotor nerve, abducens nerve, and spinothalamic tracts were uninvolved. Therefore, he was suspected to have a posterior circulation stroke. Computed tomography (CT) of the brain revealed an intracerebral hemorrhage of size 1.6 x 1.1 x 1.0 (TR X AP X CC) (CT HU+ 60) noted in the left posterior pons and left middle cerebellar peduncle, causing mild effacement of the fourth ventricle (Figure 1).

**Figure 2:**
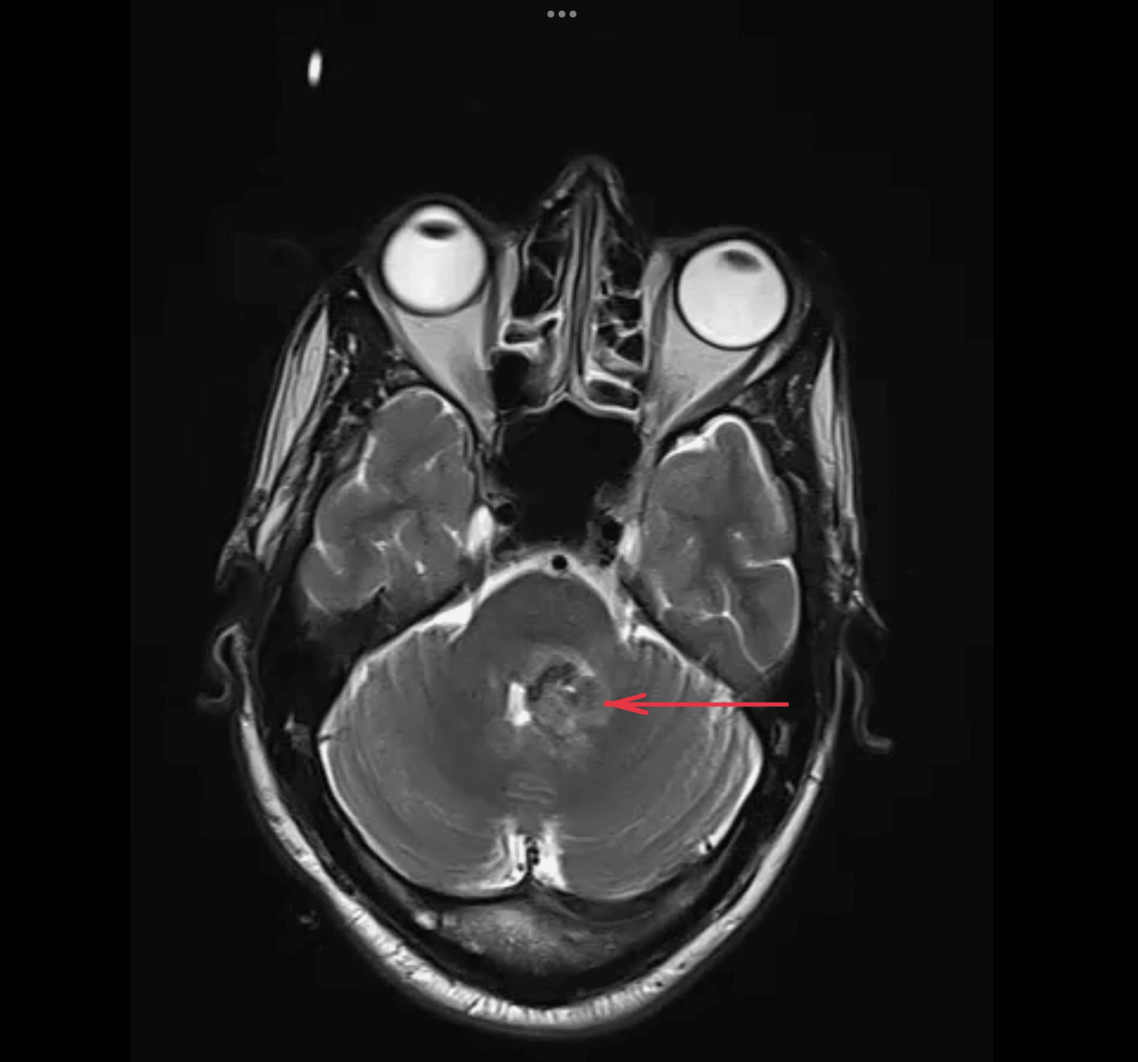
MRI brain: T2-weighted image showing heterointense area involving the left posterior aspect of the hemipons extending to left middle cerebellar peduncle and into the adjacent cerebellum, causing mass effect in the form of mild displacement and compression of fourth ventricle to the right pointed using red arrows MRI: Magnetic resonance imaging

**Figure 3:**
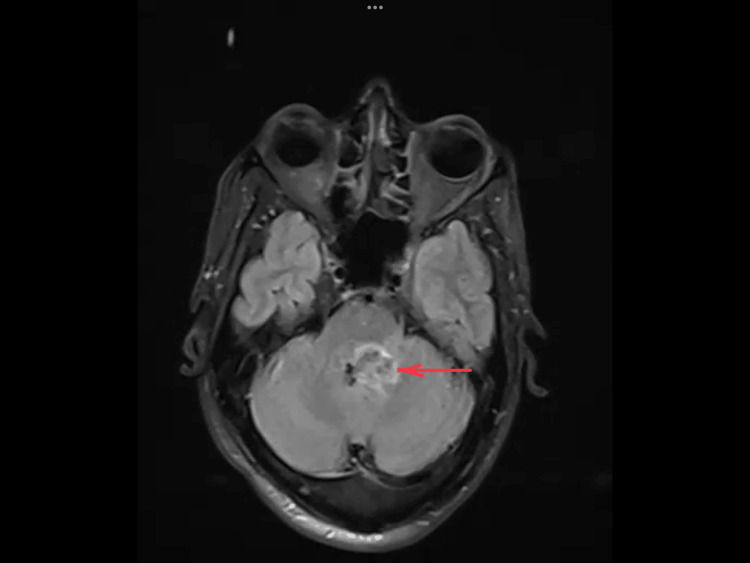
MRI brain: T2 FLAIR image showing hyperintense area involving the left posterior aspect of the hemipons extending to the left middle cerebellar peduncle and into the adjacent cerebellum, indicating hemorrhage of different stages pointed using red arrows FLAIR: Fluid-attenuated inversion recovery; MRI: magnetic resonance imaging

**Figure 4:**
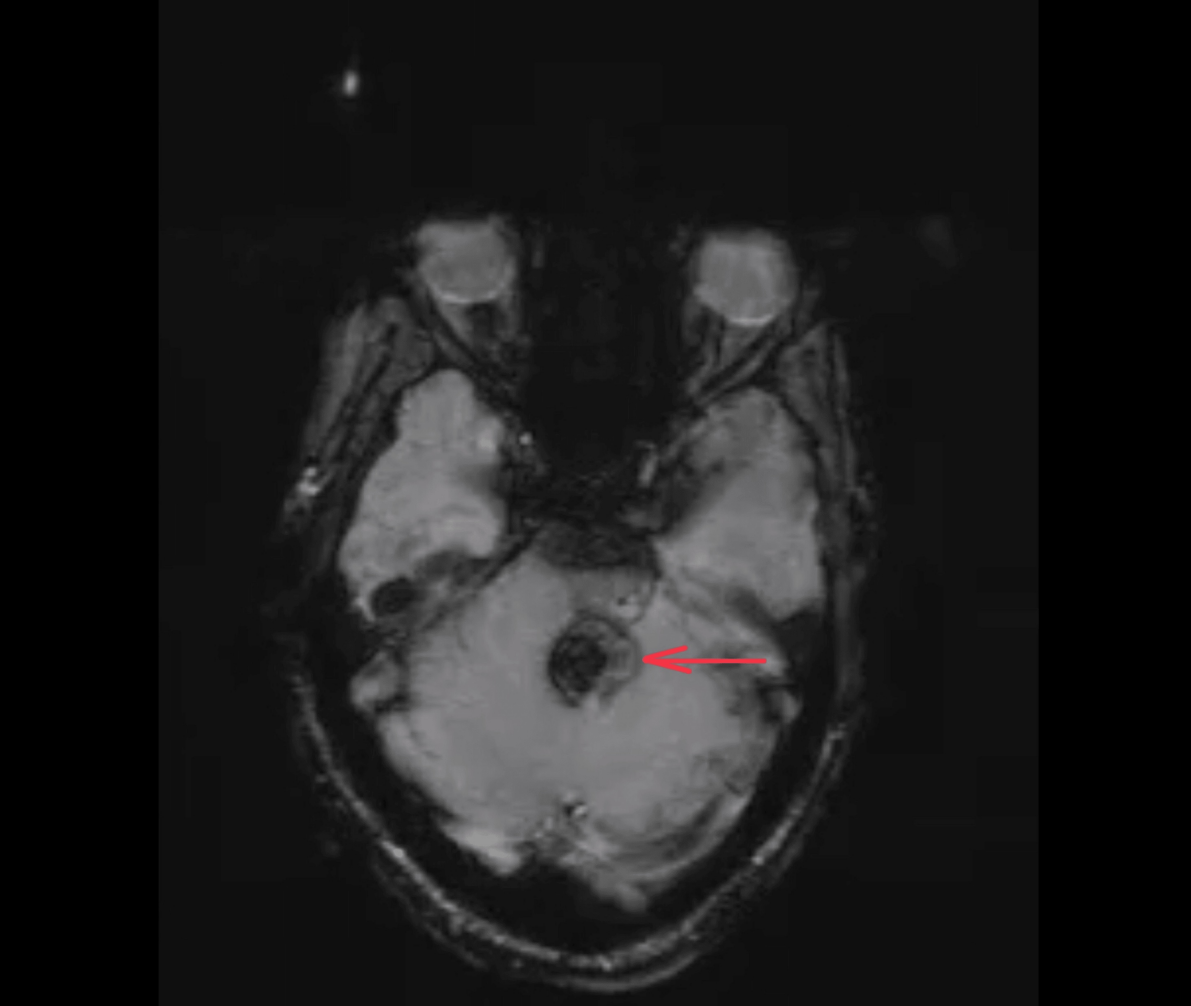
MRI brain: SWI showing areas of SWI blooming noted within likely to represent hemosiderin deposition pointed using red arrows SWI: Susceptibility weighted imaging; MRI: magnetic resonance imaging

The magnetic resonance imaging (MRI) of the brain taken revealed a hemorrhagic cavernous angioma involving the posterior aspect of the left hemipons extending to the left middle cerebellar peduncle and the adjacent cerebellum, causing a mass effect in the form of mild displacement and compression of the fourth ventricle to the right (Figures 2-4)

## Discussion

In our case, the patient presented with multiple cranial nerve palsies and cerebellum involvement as the lesion involved the pons, left middle cerebellar peduncle, and the cerebellum. In a patient with hypertension, it may be a hemorrhage due to the rupture of an aneurysm or cerebral amyloid angiopathy in an elderly individual. MRI brain was taken to determine the etiology of the hemorrhage as the patient had no predisposing conditions for intracerebral hemorrhage. MRI brain revealed cavernous angioma, an unexpected cause of hemorrhage as its incidence is low and not frequently encountered in clinical practice.

Cavernous angioma can be found incidentally or may present with clinical manifestations like seizures, headache, and neurological deficits such as limb weakness, slurred speech, and loss of balance, depending on the location of the lesion. The severity and duration of symptoms vary depending on the size, location, and number of cavernous angiomas [5]. In the case of cavernous angioma, CT brain demonstrates mass effect with perilesional edema in the presence of a recent hemorrhage [6]. The diagnosis is established through MRI. The alterations are more noticeable in T2-weighted images, which display different stages of hemorrhage [11]. To determine the number of lesions, susceptibility weighted imaging (SWI) sequences are essential. Alternative arteriovenous malformations, hemorrhagic metastases, cerebral amyloid angiopathy, chronic hypertensive encephalopathy, diffuse axonal injury (DAI), cerebral vasculitis, radiation-induced vasculopathy, and hemorrhagic primary brain tumors are among the radiographic differential diagnoses [6]. If a cavernoma is asymptomatic, conservative management is preferred, with an annual MRI as a follow-up [4]. If a single, asymptomatic cavernous malformation is located in an easily accessible, non-eloquent spot, surgical resection might be a possibility [4]. Microsurgical resection or stereotactic radiosurgery may be a viable option for alternative therapy to treat patients who present with intractable seizures, progressive neurological deterioration, hemorrhage or lesions that are not surgically accessible [4]. Research has indicated a significant risk of complications, including permanent neurological deficits, radiation-induced adverse effects, and hemorrhage recurrence following stereotactic radiosurgery [6]. The choice to intervene, however, is subjective and determined case-by-case [3].

Rho-associated protein kinase inhibitors (ROCK Inhibitors) are a promising development for medical management in the case of nonresectable cavernoma as they may slow lesion growth and minimize the development of symptoms [12].

However, neurosurgical intervention is not feasible for our patient as the lesion is located in an eloquent area of the brain that contains densely packed nuclei and cardio and respiratory centers and therefore was managed conservatively with anti-edema measures and supportive physiotherapy. The patient was counselled regarding the possibility of rebleeding which is increased, especially after a symptomatic hemorrhage. Genetic counselling is to be given for patients with multiple cavernomas, as they indicate the possibility of a familial variant. 

## Conclusions

The role of MRI brain is pivotal in determining the etiology of intracerebral hemorrhage, as various conditions like hypertensive microangiopathy, cerebral amyloid angiopathy, arteriovenous malformation, cavernous angioma, cerebral venous thrombosis, tumor, or bleeding diathesis secondary to anticoagulant therapy may present as intracerebral hemorrhage in CT brain and the management of all the above conditions differs. The incidence of cavernous angioma itself is rare, while cavernous angioma being a cause of pontine hemorrhage is even rarer. While the most common location of cavernous angioma is supratentorial, our patient had a lesion in an unusual location with an unusual presentation.

This article throws light on the need to have a wider differential when an individual with no predisposing condition or risk factors presents with an intracerebral hemorrhage. MRI brain helps deepen our understanding of the etiology of intracerebral hemorrhage while facing such challenging situations. It is, therefore, necessary to do an MRI brain scan after a CT scan to ascertain the etiology, precise location, size, and extent of the hemorrhage, as well as to prognosticate the patient.
